# The Physician and Dentist

**Published:** 1889-02

**Authors:** A. I. F. Buxbaum

**Affiliations:** Cincinnati, O.


### The Physician and Dentist.

BY A. I. F. BUXBAUM, M.D., D.D.S., CINCINNATI, O.

[A paper read before the Academy of Medicine, January 8, 1889.]

Considering the enormous number of teeth yearly sacrificed for relief from pain—estimated by some as high as twenty millions—the question of what can be done to arrest the wholesale loss presents itself for our serious consideration. Now, the medical profession look upon the loss of a tooth as a trifling matter, because they do not appreciate its present or future value. How often do we dentists hear from those who wear artificial dentures, who when young have been indifferent to the extraction of their teeth, expressions of regret at the loss of the same.

Of the inhabitants of our large cities, probably not more than 10 per cent. have a regular dentist and give even tolerable care to their teeth; while in the country not a greater portion than 1.5 per cent. ever visit the dentist's office, unless from toothache, and to have the offending member removed. When we consider the relations the deciduous teeth bear to the proper eruption of their permanent successors and their function in assisting the normal development of the bones of the face, we are impressed with the necessity of preserving the deciduous teeth until nature provides for their displacement.

The medical profession does not realize its importance, and far less so the laity. For example, a physician is treating a certain member of the family; another member, a child four and one-half, five, or six years of age, is suffering from an abscessed tooth—so-called gumboil. The parent, not knowing the value of the tooth, appeals to the attending physician, who immediately lances the abscess or orders extraction of the tooth. If the former the abscess may or may not return, but in the majority of cases it returns, the cause or irritant being still present. Now, if the physician impressed the parent that the seemingly worthless member *can* be and *should* be saved, as the successor may not appear for several years, and that a constant accumulation of pus is liable to dislodge and alter the position of the succeeding tooth, or necrosis of bone may result, and likewise that too early extraction may lead to irregularity of the teeth, I think (nine times out of ten) they would seek the advice of the dentist. Who is at fault? The physician!

Dentists discuss the great and rapid destruction of the six-year molar. It is of weaker structure than the other permanent teeth, and often decays before fairly erupted. But if the physician (when the child is six or seven years of age) would call the parent's attention to it, we could save many of the six-year-old jaw teeth. I say physician, because many of the laity do not seek a physician until caries manifest itself in some front tooth. Moreover, many do not know that the six-year molar is a permanent tooth. By saving the six-year molar we could prevent many cases of false articulation, which in itself is an irregularity. So, then, the physician has it in his power to avert many of these unsightly deformities.

There are dentists, some of whom are authorities upon dentistry, who make it their practice to extract all the six-year-old molars from both jaws and both sides, in order to give more room to the twelve-year-old molar and wisdom tooth. They claim that if the six-year molars are extracted early the wisdom teeth will be better provided for and prove to be far better teeth.

I am of the opinion that no dentist has a right to extract the six-year molars in every case that presents itself to his practice for several reasons:

First—Because nature has ever shown herself wise, and has given to man with but few exceptions thirty-two teeth. Will we not by natural selection of those teeth best adapted to give service to the human organism, bring about a change in future generations? If the six-year molar be extracted from one generation after another for generations in succession, the stamp of the six-year molar upon the human frame of each generation will be so lightly impressed that in time the six-year molar will be lost sight of. What then? Nature abhors a vacuum and provides for all space. The teeth assist in the development of the jaws. With fewer teeth there would be less development of the jaws, the jaws of future generations would be smaller, and there would have to be a universal law to extract all six-year molars, otherwise intermarriage between different nationalities, with the marked difference in size of features, would again establish irregularities! And shall we in turn go on extracting?

Again, it would take generations before the teeth would adapt themselves to that accurate articulation which they inherit, so to speak, from nature. They are not trees that we can prop up with sticks when we please. Their growth in a certain direction is given them from the start.

Second—Suppose the six-year molars being extracted and more room and nourishment being afforded the wisdom teeth, and the latter do develop superior in quality to the six-year molars, will their *position* offer more service to the human economy, and will it have been a wise operation to have extracted the six-year molars? Do we not seek the aid of the six-year and twelve-year molars for our heavy crushing and grinding, not because the six-year molar is stronger, but on account of its position? Looking at the matter from this standpoint, I say, let us take care of the six-year molars for reasons stated above, and not less so for the third reason, namely, that we can not foretell what teeth will have to be sacrificed, notwithstanding all care, as we advance from childhood to old age.

I do not intend to traverse the field of irregularity and give its many causes and methods of correction, but only touch upon that part of the ground where those before me to-night can prove a valuable aid in assisting the dentist in preventing deformities.

I find that to a certain degree, again, it is in the power of physicians to prevent many of those cases of acquired irregularity. How many children, as I have said before, are there who, at the tender age of four and a half to five and six years, or even seven, present themselves to the dentist? I will answer, comparatively few, although the tendency to do so on the part of the parents is greater now than formerly. And who sees the child at that tender age? I answer, the family physician, and it is as much his duty to see that the oral cavity of that innocent one is doing well as it is to care for any other vital organ. But he doesn't! Why? Because there are many who do not know which are the permanent teeth and which the temporary; when nature is failing to do her duty and when she is not; and what constitutes an irregularity.

What does constitute an irregularity? In answer to the above I quote Prof. Guilford, one of our authorities upon orthodontia, or dental irregularities. He speaks of two general kinds, one where certain individual teeth (one or many) stand out of line, or are imperfectly placed in line, but where those still in place describe the normal outline of the particular jaw; the other where their outline is so changed from a normal standard as to constitute a deformity or malformation. Either of these or a combination of the two is what generally calls for the interference of the dental practitioner.

Irregularities as to their origin may be either hereditary or acquired, the one resulting from causes operating before the birth of the individual or the eruption of the teeth, and the other from circumstances attending their eruption or subsequent to it. I look upon heredity as a powerful factor in establishing deformities, and not less so that of the teeth; and I regard all cases where heredity is the cause to be more difficult to correct than where irregularity results from other causes, because in the former we have not alone the irregularity to remove, but also that *tendency* towards irregularity.

I have seen many families where not only the form but also where the structure of the teeth were the same in the individual members, and caries attacked the same spots and assumed the same outline in the individual members, showing the power of heredity. The child may inherit some peculiarities from one parent and some from the other; this being the case, if the parents be of different nationalities or if there be a marked difference in size and feature, the one having large teeth and large jaws, and the other small teeth and small jaws, it is very likely that the child may inherit the small jaw of the one and the large teeth of the other, the disparity between the two resulting in malposition of the teeth from insufficient room to accommodate them. This is generally believed to be a frightful source of irregularity. Nature has a strong tendency to harmonize, and she will do a great deal to bring about harmony, but her efforts are not always successful, and occasionally a little advice from the family physician, followed up by a little interference on the part of a conscientious dentist, will do a great deal.

Correct facial expression and harmony of feature are far more important to the female than to the male, and if the attending physician could point out the results of an irregularity, such as distortion of features, interference with speech, superinducement of caries, and injury to health resulting from inefficient mastication, there would be less cases of deformity walking our streets.

The hygiene of the oral cavity is a matter that should interest every one. The prevention of disease is to be not only the principal work of the medical man in the future, but will claim the largest share of the attention of the dentist in years to come. The people must be taught the value of antisepsis; that in proportion as they keep the mouth pure will the whole organism be more free from disease. And they should be taught at an *early* age. And who sees the child at an early age? The physician. By teaching hygienic dentistry to our patients, impressing upon them that by the preservation of the teeth they not only retain the natural expression of the face, but assist largely in promoting the health and strength of the entire organism, and that proper mastication is necessary to the maintenance of health, will we accomplish good results.

And let me say right here: As long as there is one jaw tooth, sensitive, decayed, and giving trouble, so long will that side of the jaw fail to give satisfaction in mastication. How many cases of dyspepsia are treated daily by our best physicians where the mouth is full of diseased teeth.

In treating dyspepsia every physician should inquire into the condition of the mouth, see that the teeth are filled, bad members extracted and artificial dentures inserted, and all tartar removed. Many a person acquires dyspepsia from the use of one side of the mouth, when nature ordained that both sides were necessary to accomplish the desired result.

How many cases of neuralgia are due to the teeth? Do all physicians stop to look into the condition of the teeth when a case of facial neuralgia presents itself?

A carious tooth, chronic inflammation of the pulp of a tooth, difficult eruption of a wisdom tooth may cause facial neuralgia. *Apropos* a case occurs to me, where in my practice some time ago a young lady was treated by her physician for facial neuralgia, but got no relief. She applied to her dentist, who failed to find the trouble. She then called at my office, and, upon examination I found the gum swollen and glossy behind the second right inferior molar, and with an exploring needle felt the wisdom tooth. I lanced it and her neuralgia shortly disappeared.

Another cause, and which is often the case, was due to a large cavity on posterior surface of wisdom tooth. Patient complained of neuralgia in the neck, head, and face. This was relieved by filling the tooth. Erosion of the teeth is a frequent cause.

Many people think they are suffering from catarrah and derangement of the digestive system on account of disagreeable odor and bad taste in the mouth, and apply to the physician; these symptoms if investigated, would be found to be due to carious teeth. This condition does not result where hygienic dentistry is observed, and in closing let me once more make a final appeal to you now present, to see to the saving of the six-year molars, and the correction of irregularities of innocent ones, that in after years deformity may not stare them in the face.

